# Neonatal frontal-limbic connectivity is associated with externalizing behaviours in toddlers with Congenital Heart Disease

**DOI:** 10.1016/j.nicl.2022.103153

**Published:** 2022-08-17

**Authors:** Alexandra F. Bonthrone, Andrew Chew, Megan Ní Bhroin, Francesca Morassutti Rech, Christopher J. Kelly, Daan Christiaens, Maximilian Pietsch, J-Donald Tournier, Lucilio Cordero-Grande, Anthony Price, Alexia Egloff, Joseph V. Hajnal, Kuberan Pushparajah, John Simpson, A. David Edwards, Mary A. Rutherford, Chiara Nosarti, Dafnis Batalle, Serena J. Counsell

**Affiliations:** a Centre for the Developing Brain, School of Biomedical Engineering & Imaging Sciences, King’s College London, London, UK; b Trinity College Institute of Neuroscience and Cognitive Systems Group, Discipline of Psychiatry, School of Medicine, Trinity College, Dublin, Ireland; c Department of Electrical Engineering (ESAT/PSI), KU Leuven, Leuven, Belgium; d Department for Forensic and Neurodevelopmental Sciences, Institute of Psychiatry, Psychology and Neuroscience, King’s College London, London, UK; e Biomedical Image Technologies, ETSI Telecomunicación, Universidad Politécnica de Madrid & CIBER-BBN, Madrid, Spain; f Biomedical Engineering Department, School of Biomedical Engineering and Imaging Sciences, King’s College London, London, UK; g Paediatric Cardiology Department, Evelina London Children's Healthcare, London, UK; h Department of Child and Adolescent Psychiatry, Institute of Psychiatry, Psychology and Neuroscience, King's College London, London, UK

**Keywords:** Congenital Heart Disease, Neonatal, Diffusion-weighted MRI, Graph theory, Externalizing, Internalizing

## Abstract

Children with Congenital Heart Disease (CHD) are at increased risk of neurodevelopmental impairments. The neonatal antecedents of impaired behavioural development are unknown. 43 infants with CHD underwent presurgical brain diffusion-weighted MRI [postmenstrual age at scan median (IQR) = 39.29 (38.71–39.71) weeks] and a follow-up assessment at median age of 22.1 (IQR 22.0–22.7) months in which parents reported internalizing and externalizing problem scores on the Child Behaviour Checklist. We constructed structural brain networks from diffusion-weighted MRI and calculated edge-wise structural connectivity as well as global and local brain network features. We also calculated presurgical cerebral oxygen delivery, and extracted perioperative variables, socioeconomic status at birth and a measure of cognitively stimulating parenting. Lower degree in the right inferior frontal gyrus (partial ρ = −0.687, p < 0.001) and reduced connectivity in a frontal-limbic sub-network including the right inferior frontal gyrus were associated with higher externalizing problem scores. Externalizing problem scores were unrelated to neonatal clinical course or home environment. However, higher internalizing problem scores were associated with earlier surgery in the neonatal period (partial ρ = −0.538, p = 0.014). Our results highlight the importance of frontal-limbic networks to the development of externalizing behaviours and provide new insights into early antecedents of behavioural impairments in CHD.

## Highlights

• Diffusion MRI network analyses in babies with congenital heart disease.
• Internalizing and externalizing behaviors assessed at 22 months.
• Lower frontal-limbic connectivity associated with higher externalizing scores.
• Lower R inferior frontal gyrus degree associated with higher externalizing scores.
• Earlier age at surgery associated with higher internalizing behaviours.

## 1 Introduction

Congenital heart disease (CHD) occurs in up to 1 % of births (EUROCAT, 2015). Over 90 % of infants with CHD survive into adulthood (Wren and Sullivan, 2001), yet survivors are at increased risk of difficulties across several domains including executive functioning, cognition, and internalizing and externalizing behaviors (Clancy et al., 2020, Feldmann et al., 2021, Marino et al., 2012). These difficulties may persist into adolescence (Bellinger et al., 2011) and adulthood (Klouda et al., 2017) and have a prolonged impact on quality of life (Dewey and Volkovinskaia, 2018) and educational achievement (Lawley et al., 2019). As such, understanding the mechanisms underpinning impaired neurodevelopment in survivors of CHD is critical.

MRI studies have identified both acquired brain lesions (Beca et al., 2013, Kelly et al., 2019a, Miller et al., 2007, Stegeman et al., 2021) and impaired brain development in infants with CHD. In particular, brain volumes are reduced (Bouyssi-Kobar et al., 2012, Limperopoulos et al., 2010, Ng et al., 2020, von Rhein et al., 2015) and development of the cortex is impaired compared to healthy controls (Kelly et al., 2017, Kelly et al., 2019b). The degree of altered early brain development is associated with reductions in cerebral oxygen delivery (CDO_2_) (Kelly et al., 2017, Kelly et al., 2019b, Sun et al., 2015).

Recently, studies have reported neonatal neuroimaging correlates of impaired cognition in children with CHD. In early childhood, lower cognition has been associated with reduced cortical and cerebellar volumes (Meuwly et al., 2019) and dilated CSF spaces (Heye et al., 2018, Knirsch et al., 2017) after surgery. 6 year old children with CHD and IQ scores below 85 also had lower postoperative neonatal basal ganglia, thalamus and brainstem volumes compared with survivors with higher IQ scores (Claessens et al., 2018). In addition, we have previously shown that reduced CDO_2_ is indirectly associated with lower cognitive scores in early childhood through the mediating effect of impaired presurgical neonatal deep grey matter development (Bonthrone et al., 2021a). Diffusion MRI (dMRI) has been used to investigate both brain development in neonates (Claessens et al., 2019, Karmacharya et al., 2018, Kelly et al., 2019b, Mulkey et al., 2014) and neural correlates of cognitive abilities in adolescents and adults (Brewster et al., 2015, Ehrler et al., 2020, Ehrler et al., 2021, Rollins et al., 2014) with CHD. However, the neonatal dMRI correlates of childhood outcomes and the neonatal neuroimaging correlates of internalizing and externalizing symptoms in CHD have yet to be fully characterized.

Graph theory encompasses a set of analyses designed to describe global and local organizational properties of brain networks extracted from MRI data (Bullmore and Sporns, 2009). In infants with CHD, reduced functional connectivity in a cortico-subcortical sub-network (De Asis-Cruz et al., 2018) and altered structural network topology (Schmithorst et al., 2018) have been reported before surgery as well as reduced network integration and greater network segregation pre- and postoperatively (Feldmann et al., 2020). We have previously reported intact global network topology but reduced structural brain connectivity in a sub-network encompassing the cerebellum, deep grey matter, temporal lobe and parieto-occipital regions before surgery, compared to healthy controls (Ní Bhroin et al., 2020). In a mixed cohort of infants with CHD and those with hypoxic-ischemic encephalopathy, reduced neonatal global efficiency, a measure of network integration, was associated with poorer motor abilities at 30 months (Ramirez et al., 2022). Graph theoretical approaches have been used to describe changes in global and local brain networks associated with internalizing disorders such as depression and anxiety (Ely et al., 2021), externalizing disorders such as ADHD (Beare et al., 2017, Li et al., 2021), and internalizing and externalizing behaviors in children born prematurely (Gilchrist et al., 2022). However, to our knowledge no study has characterized the relationship between neonatal brain network organization and behavioral outcomes in children with CHD.

In this study we aimed to investigate the relationship between neonatal presurgical global and local structural network features extracted from multi-shell diffusion MRI, clinical and environmental measures, and behavioral outcomes at 22 months in infants with CHD.

## 2 Materials and methods

### 2.1 Ehical approval

The National Research Ethics Service West London committee provided ethical approval (07/H0707/105). In accordance with the declaration of Helsinki, informed written parental consent was obtained before neonatal MRI and neurodevelopmental follow-up at 22 months.

### 2.2 Recruitment

Infants with critical or serious CHD were recruited between 2016 and 2019 at St Thomas’ Hospital London. Based on a previously published UK categorization (Ewer et al., 2011) critical CHD was defined as infants with hypoplastic left heart syndrome (HLHS), interrupted aortic arch, pulmonary atresia with an intact ventricular septum, transposition of the great arteries (TGA) and all infants requiring surgery within the first 28 days of life with the following conditions: aortic stenosis, coarctation of the aorta (CoA), pulmonary stenosis, pulmonary atresia with ventricular septal defect, tetralogy of Fallot (TOF) and total anomalous pulmonary venous connection. Serious CHD was defined as any cardiac lesion not defined as critical, which requires cardiac catheterization or surgery between 1 month and 1 year of age. Infants were categorized into abnormal streaming of blood, left-sided heart abnormalities and right-sided heart abnormalities according to the haemodynamic impact of their anatomy using the sequential segmental approach (Anderson et al., 1984). Exclusion criteria included suspected or confirmed chromosomal abnormality or congenital syndrome, previous neonatal surgery before recruitment (excluding cardiac catheterization procedures) or suspected congenital infection (Kelly et al., 2019a).

### 2.3 MRI acquisition

Brain MRI was performed during natural sleep on a Philips (Best, Netherlands) 3 Tesla system situated in the neonatal intensive care unit at St. Thomas’ Hospital using a 32-channel neonatal head coil and neonatal positioning device (Hughes et al., 2017). Earplugs moulded from silicone-based putty were placed in the external auditory meatus (President Putty, Coltene Whaledent, Mahwah, NJ), followed by neonatal earmuffs (MiniMuffs, Natus Medical Inc, San Carlos, CA) and an acoustic hood placed over the infant. Scanning was supervised by a paediatrician trained in MR procedures and pulse oximetry, temperature, electrocardiography, and respiratory rate were monitored throughout.

T1-weighted (magnetisation prepared rapid gradient echo, repetition time (TR)/echo time (TE) 11/4.6 ms, flip angle 9°, voxel size 0.8 mm^3^), T2-weighted (multislice turbo spin echo TR/TE 12000/156 ms, in-plane resolution 0.8 × 0.8 mm, slice thickness 1.6 mm, 0.8 mm overlap) and susceptibility-weighted imaging (TR/TE 3200/25 ms, flip angle 12°, voxel size 0.45 × 0.45 × 1.8 mm) were acquired. dMRI was acquired with a high angular resolution diffusion multi-shell protocol designed for the neonatal brain (TR/TE 3800/90 ms, multiband factor 4, sensitivity encoding in-plane acceleration factor 1.2, in-plane resolution 1.5 × 1.5 mm, slice thickness 3 mm, 1.5 mm overlap, 300 volumes, diffusion gradient encoding: b = 0 s/mm^2^ (n = 20), b = 400 s/mm^2^ (n = 64), b = 1000 s/mm^2^ (n = 88), b = 2600 s/mm^2^ (n = 128) with 4x interleaved phase encoding) (Hutter et al., 2018). Quantitative flow imaging was acquired using velocity sensitized phase contrast angiography (PCA) with a single-slice T1-weighted fast field echo sequence (TR/TE 6.4/4.3 ms, flip angle 10°, repetitions 20, velocity encoding 140 cm/s, field of view 100 × 100 mm, resolution 0.6 × 0.6 × 4.0 mm) (Varela et al., 2012). PCA was available in 37 infants (not acquired due to infant waking N = 3; unsuitable for analysis N = 3).

### 2.4 MR image review

All images were reviewed by two perinatal neuroradiologists and lesions were recorded as white matter injury (WMI), cerebellar haemorrhage or intraventricular haemorrhage as reported previously (Kelly et al., 2019a). WMI was classified into normal (no injury), mild (≤3 foci and all ≤2 mm), moderate (>3 and ≤ 10 foci or any >2 mm) or severe (>10 foci) (Kelly et al., 2019a).

Overall, each infant was categorized into two brain injury groups: normal/mild (mild: intraventricular haemorrhage, and/or cerebellar hemorrhage ≤2 mm, and/or mild WMI) and moderate/severe (moderate: cerebellar hemorrhage >2 mm and/or moderate WMI; severe: severe WMI) (Bonthrone et al., 2021b, Kelly et al., 2019a). Brain injury ratings are summarized in Table 2.

### 2.5 MRI pre-processing

T2-weighted images acquired in the axial and sagittal plane underwent motion correction and reconstruction to a 0.5 mm grid (Cordero-Grande et al., 2018). dMRI underwent parallel imaging reconstruction, denoising (Cordero-Grande et al., 2019, Veraart et al., 2016), Gibbs ringing artefact suppression (Kellner et al., 2016), and correction for motion and image distortion using Spherical Harmonics and Radial Decomposition reconstruction to a 1.5 mm grid (Christiaens et al., 2021).

### 2.6 Structural network construction

Reconstructed T2-weighted images were bias field corrected (Tustison et al., 2010), brain extracted (Smith, 2002), and segmented into tissue classes [grey matter, white matter, extracerebral cerebral spinal fluid (CSF), ventricles, deep grey matter, brainstem, hippocampus and amygdala, and cerebellum] using an automatic neonatal-specific segmentation algorithm that employs expectation maximization (Makropoulos et al., 2014, Makropoulos et al., 2018).

A neonatal adaptation (Shi et al., 2011) of the automated anatomical labelling (AAL) parcellation (Tzourio-Mazoyer et al., 2002) consisting of 93 cortical, subcortical and cerebellar regions was registered to each infant’s T2-weighted image using the diffeomorphic symmetric image normalization method in Advanced Normalization Tools (Avants et al., 2011). Each infant’s tissue maps and parcellations were registered to their dMRI (average B = 0 vol as the target) using rigid registration in the Image Registration Toolkit (Studholme et al., 1999).

Orientation distribution functions (ODFs) for tissue and free water were estimated using multi-shell multi-tissue constrained spherical deconvolution in MRtrix3 (Jeurissen et al., 2014, Tournier et al., 2019), with fixed tissue response functions for white matter and CSF (Pietsch et al., 2019) averaged from control infants imaged using the same protocol (used in Ní Bhroin et al, see (Ní Bhroin et al., 2020) for details). ODFs were normalized to obtain quantitative measures (Dhollander et al., 2021, Raffelt et al., 2017, Tournier et al., 2019). Probabilistic tractography was used to generate 10 million streamlines from the tissue ODFs with anatomically constrained probabilistic tractography (Smith et al., 2012) and biologically accurate weights (SIFT2) (Smith et al., 2015, Tournier et al., 2019). Structural connectivity networks were constructed for each infant with regions of the AAL parcellation as nodes and sum of SIFT2-weighted streamlines connecting each region as edges.

### 2.7 Graph theory feature extraction

Graph theory features were calculated using the Brain Connectivity Toolbox (Rubinov and Sporns, 2010) in Matlab R2020b. Network infrastructure was assessed with network density defined as the proportion of observed edges out of all possible edges (Kaiser, 2011) and nodal strength defined as the sum of edge weights connected to a node. Average nodal strength was also calculated. Network integration was assessed with global efficiency defined as the inverse of the average shortest path length between nodes (Achard et al., 2007) and nodal degree defined as the number of connections linking each node to the rest of the network. Network segregation was assessed with nodal efficiency, defined for each node as the inverse of the average shortest path length connecting all neighbours of that node; and local efficiency, defined as the average nodal efficiency across all nodes of the network (Bullmore and Sporns, 2009, Rubinov and Sporns, 2010).

Variance in network features may be confounded by differences in network density between infants. In order to adjust for this effect, we employed a cost-correction approach (Achard et al., 2007, Batalle et al., 2017). For each infant, a series of networks were created by thresholding original reconstructed networks to set densities from 0.05 to 0.5 increasing in 0.01 steps (proportional thresholding). Nodal strength, nodal degree as well as global, local and nodal efficiency were extracted at each network density and averaged across the range of cost corrections, resulting in a “cost-corrected” measure of each feature (Batalle et al., 2017, Ginestet et al., 2011).

We partitioned nodes in structural networks into two distinct groups corresponding to a core and periphery structure using an adapted version of the Kernighan-Lin algorithm for graph partitioning (Borgatti and Everett, 2000, Newman, 2006) as we have previously in a larger group of infants with CHD and controls (Ní Bhroin et al., 2020). In keeping with previously published results (Ní Bhroin et al., 2020), nodes partitioned into 34 core and 59 peripheral regions (Supplementary Table 1). Edges were characterized as core (connecting core nodes), peripheral (connecting peripheral nodes) or feeder (connecting a core and a peripheral node).

### 2.8 Cerebral oxygen delivery

Cerebral blood flow was quantified from phase-contrast angiography using previously published methods (Kelly et al., 2017, Kelly et al., 2019b).

#### 2.8.1 Neurodevelopmental assessment

All infants attended a follow-up assessment at a median (IQR) age of 22.10 (21.95–22.36) months. Infants completed the Bayley Scales of Infant and Toddler Development–Third Edition (Bayley, 2006) administered by a developmental paediatrician to obtain cognitive composite scores [test mean (SD) = 100 (15)]. In addition, parents completed the Child Behavior Checklist (CBCL) 1.5–5 questionnaire (Achenbach and Rescorla, 2000) and internalizing (comprised of emotionally reactive, anxious/depressed, somatic complaints and withdrawn subscales) and externalizing (comprised of attention problems and aggression subscales) raw problem scores and *T*-scores [test mean (SD) = 50 (10)] were calculated (higher scores indicate increased symptomatology). Internalizing and externalizing problem scores were also categorised into normal (*T*-score < 60), borderline (*T*-score 60–63) and clinical (indicating a score that is within the same range as scores obtained from children referred for internalizing/externalising problems; *T*-score > 63)(Achenbach and Rescorla, 2000).

#### 2.8.2 Cognitively stimulating parenting

At follow-up assessment parents completed the cognitively stimulating parenting scale (CSPS) (Wolke et al., 2013), a 21‐item questionnaire adapted from the Home Observation for Measurement of the Environment (HOME) Inventory (Bradley and Caldwell, 1984) designed to assess the level of cognitive stimulation at home (CSPS score range 0–46). Details of this questionnaire have been published previously (Bonthrone et al., 2021b).

#### 2.8.3 Socioeconomic status

Index of multiple deprivation (IMD) was obtained from maternal postcode recorded at birth. IMD is a composite measure of socioeconomic status in England encompassing factors related to income, employment, education, health, and crime (https://imd-by-postcode.opendatacommunities.org/). IMD was obtained from the 2015 data release and reported as scores and quintiles (most to least deprived). It was not possible to obtain IMD for one infant.

### 2.9 Clinical information

Hospital records were reviewed to calculate days on the intensive care unit (ICU) post-surgery, time on bypass during surgery, and days to corrective or final palliative surgery. In children who underwent more than one surgery, days on ICU and time on bypass were summed across procedures (Bonthrone et al., 2021b).

#### 2.9.1 Statistical analysis

Standardized residuals were calculated for raw internalizing and externalizing scores adjusting for corrected age at follow-up assessment. Age adjusted raw scores were used for all subsequent analyses. A Kruskal-Wallis H test was used to assess the relationship between CHD subgroup and internalizing and externalizing scores. Kruskal-Wallis was used due to uneven group sizes (Abnormal streaming of blood n = 22; Right heart abnormalities n = 12; Left heart abnormalities n = 9).

Partial Spearman’s rank correlations were used to assess the association between internalizing and externalizing scores, clinical and environmental features: CDO_2_, days in intensive care, time on bypass and days to surgery, CSPS and IMD; and graph theory features: total network density, cost-corrected average nodal strength, global efficiency and local efficiency, cost-corrected nodal efficiency, strength and degree for each node.

All analyses covaried for gestational age at birth (GA), sex, brain injury severity, cognitive composite score and IMD (except in the correlation between IMD and internalizing/externalizing problem scores). Postmenstrual age at MRI (PMA) was also included as a covariate in the CDO_2_ and graph theory features analyses.

All p-values underwent false discovery rate correction (p_FDR_) to correct for multiple comparisons (Benjamini and Hochberg, 1995).

#### 2.9.2 Network based statistics

Edge-wise structural connectivity was assessed with the network-based statistics (NBS) toolbox, which implements permutation testing to detect edge-wise associations with brain connectivity (Zalesky et al., 2010). A general linear model with 10,000 permutations and cluster-based family-wise error rate correction for multiple comparisons was used to test for associations between network extent (i.e., total number of connections) and internalizing and externalizing symptoms. The critical p-value was set at 0.025. A test-statistic threshold (*t* = 3.1) was set, where connections exceeding this threshold were considered significant. NBS results are highly dependent on the primary test-statistic threshold, therefore we additionally tested a range of values (*t* = 2.5–3.5, in 0.1 steps). Brain injury rating, cognitive composite score, sex, GA, PMA and IMD were entered as covariates. Significant networks were visualized with BrainNet Viewer (Xia et al., 2013).

### 2.10 Data availability

The anonymised processed data analysed during this study is available from the corresponding author upon reasonable request.

## 3 Results

### 3.1 Sample characteristics

**Fig. 1:**
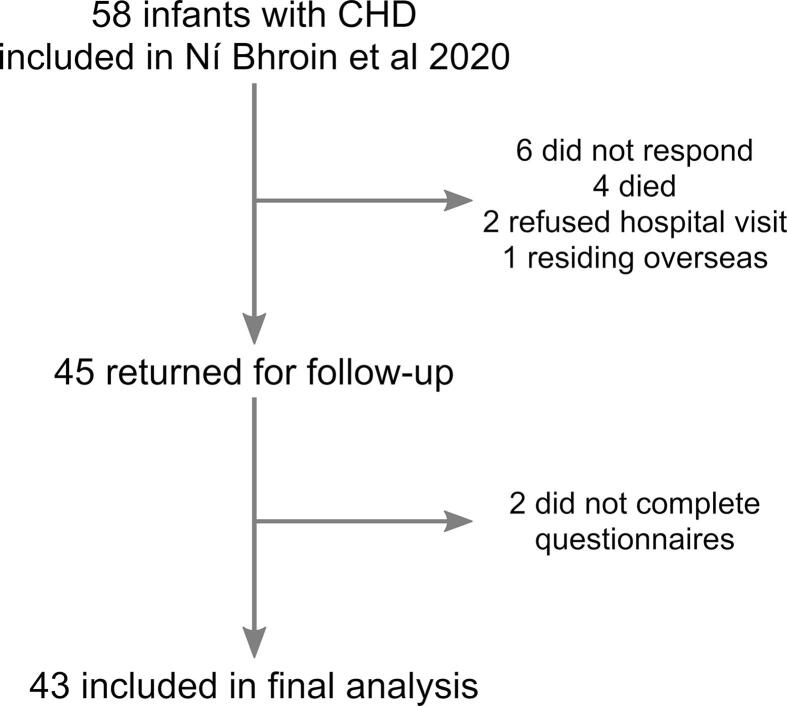
Flow diagram of inclusion of infants from Ni Bhroin and colleagues (Ní Bhroin et al., 2020) in the current study.

A prospective cohort of 43 infants with critical or serious CHD was recruited between 2016 and 2019 at St Thomas’ Hospital London (Table 1). The infants were part of a larger group (N = 58) included in our previous publication investigating differences in graph theoretical measures in infants with CHD and controls (Ní Bhroin et al., 2020), details of inclusion are given in Fig. 1.

**Table 1:** Sample Demographics.

Measure	N = 43
Gestational Age at Birth, Median (IQR)	38.57 (38.21–38.86)
Postmenstrual Age at Scan, Median (IQR)	39.29 (38.71–39.25)
Male, N (%)	21 (49.8)
Primary Cardiac Lesion	
Abnormal Streaming of Blood	
Transposition of the Great Arteries, N (%)	22 (51.2)
Left-sided heart abnormalities	
Coarctation of the Aorta, N (%)	8 (18.6)
Hypoplastic Left Heart Syndrome, N (%)	1 (2.3)
Right-sided heart abnormalities	
Tetralogy of Fallot, N (%)	4 (9.3)
Pulmonary Stenosis, N (%)	4 (9.3)
Pulmonary Atresia, N (%)	3 (7)
Tricuspid Atresia, N (%)	1 (2.3)
Minutes on bypass median (IQR)	143 (72–189)
Days to corrective or final palliative surgery median (IQR)	15 (10–144)
Days on Intensive Care Unit post-surgery median (IQR)	5 (3–7)

**Table 2:** Brain injury ratings in infants with CHD.

None/Mild, N (%)	37 (86.0)
Moderate/Severe, N (%)	6 (14.0)
Brain Injury Type	
None, N (%)	27 (62.8)
Mild WMI, N (%)	8 (18.6)
Mild cerebellar haemorrhage, N (%)	2 (4.6)
Moderate WMI with mild cerebellar haemorrhage, N (%)	1 (2.3)
Moderate WMI, N (%)	4 (9.4)
Severe WMI, N (%)	1 (2.3)

### 3.2 Neurodevelopmental outcome scores

Internalizing and externalizing problem scores, as well as cognitive composite, cognitively stimulating parenting scale (CSPS) scores and index of multiple deprivation (IMD) scores in toddlers with CHD are summarized in Table 3. Internalizing behaviors were categorised as clinical in 7 % of infants (Left heart abnormalities n = 2, right heart abnormalities n = 1). 10 % of infants were categorised as clinical on the externalizing scale (Abnormal Streaming of Blood n = 2, Left heart abnormalities n = 1, right heart abnormalities n = 4). 28 % of infants (n = 12; Abnormal Streaming of Blood n = 4, Left heart abnormalities n = 3, right heart abnormalities n = 4) scored in the borderline or clinical ranges for internalizing or externalizing problem scores.

**Table 3:** Neurodevelopmental outcome scores in infants with CHD.

Internalizing raw score Median (IQR)	7 (3–11.5)
Internalizing *T*-score Mean (SD)	48.2 (10.6)
Normal (<60) N (%)	34 (79)
Borderline (60–63) N (%)	6 (14)
Clinical (>63) N (%)	3 (7)
Abnormal Streaming of Blood Internalizing *T*-score Median (IQR)	48 (38–54.5)
Left heart abnormalities Internalizing *T*-score Median (IQR)	49 (41–62)
Right heart abnormalities Internalizing *T*-score Median (IQR)	49 (40–57)
Externalizing raw score median (IQR)	12 (8–18)
Externalizing *T*-score mean (SD)	50.3 (9.5)
Normal (<60) N (%)	38 (88)
Borderline (60–63) N (%)	1 (2)
Clinical (>63) N (%)	4 (10)
Abnormal Streaming of Blood Externalizing *T*-score Median (IQR)	50 (46–56.75)
Left heart abnormalities Externalizing *T*-score Median (IQR)	50 (40–57)
Right heart abnormalities Externalizing *T*-score Median (IQR)	47.5 (43.75–55.75)
Cognitive Composite Score Mean (SD)	93.6 (10.2)
Cognitively Stimulating Parenting Scale Mean (SD)	32.0 (6.6)
Index of Multiple Deprivation Median (IQR)	18.66 (13.18–30.27)
Index of Multiple Deprivation Quintile	
1^st^ (Most deprived)	8
2	11
3	9
4	9
5	5

### 3.3 Clinical and environmental correlates of internalizing and externalizing problem scores

**Fig. 2:**
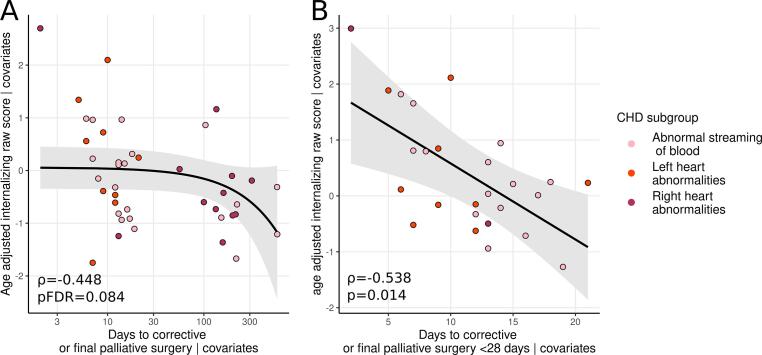
Relationship between days to corrective or final palliative surgery and age adjusted internalizing raw scores in A) the whole sample, B) infants who underwent corrective or final palliative surgery at <28 days. Covarying for gestational age at birth, sex, brain injury rating, cognitive composite score and IMD.

Although the relationship between internalizing scores and time to surgery was not significant (partial ρ = −0.448 p_FDR_ = 0.084, covarying for gestational age at birth (GA), sex, brain injury severity, cognitive composite score and IMD; Fig. 2A), post-hoc tests revealed this relationship was significant (partial ρ = −0.538, p_uncorrected_ = 0.014; Fig. 2B) in infants who underwent final corrective or palliative surgery when they were younger than 28 days (n = 27).

A post-hoc analysis of age adjusted raw items that are used to obtain internalizing problem scores, revealed days to surgery was correlated with emotionally reactive (partial ρ = −0.522 p_FDR_ = 0.036), and somatic complaints (partial ρ = −0.570 p_FDR_ = 0.036) but not withdrawn (partial ρ = −0.447 p_FDR_ = 0.064) or anxiety and depression (partial ρ = −0.289 p_FDR_ = 0.217) subscales.

There were no additional significant relationships between clinical or environmental variables and age adjusted raw internalizing or externalizing problem scores (Table 4).

**Table 4:** Relationship between age adjusted internalizing and externalizing raw scores and clinical variables.

	Internalizing	Externalizing
CHD subgroup	H = 0.394, p_FDR_ = 0.958	H = 0.152, p_FDR_ = 0.958
CDO_2_	Partial ρ = −0.073, p_FDR_ = 0.958	Partial ρ = −0.106, p_FDR_ = 0.958
Minutes on bypass	Partial ρ = −0.119, p_FDR_ = 0.958	Partial ρ = 0.211, p_FDR_ = 0.958
Days on ICU post-surgery	Partial ρ = −0.105, p_FDR_ = 0.958	Partial ρ = 0.037, p_FDR_ = 0.958
Days to corrective or final palliative surgery	Partial ρ = −0.448, p_FDR_ = 0.084	Partial ρ = −0.170, p_FDR_ = 0.958
IMD	Partial ρ = 0.086, p_FDR_ = 0.958	Partial ρ = −0.009, p_FDR_ = 0.958
CSPS	Partial ρ = −0.021, p_FDR_ = 0.958	Partial ρ = −0.296, p_FDR_ = 0.553

### 3.4 Nodal brain network correlates of internalizing and externalizing problem scores

**Fig. 3:**
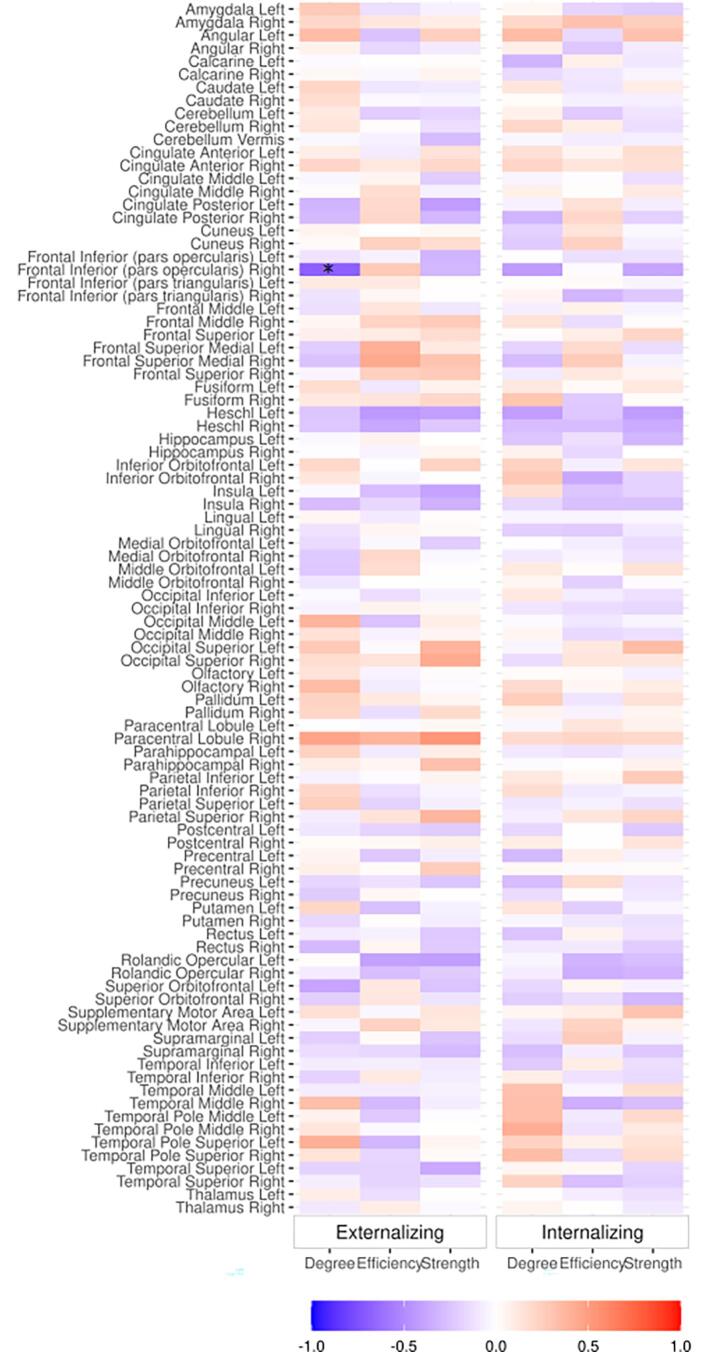
Partial Spearman’s rank correlation coefficients between nodal characteristics and age adjusted internalizing and externalizing raw scores adjusted for covariates.

**Fig. 4:**
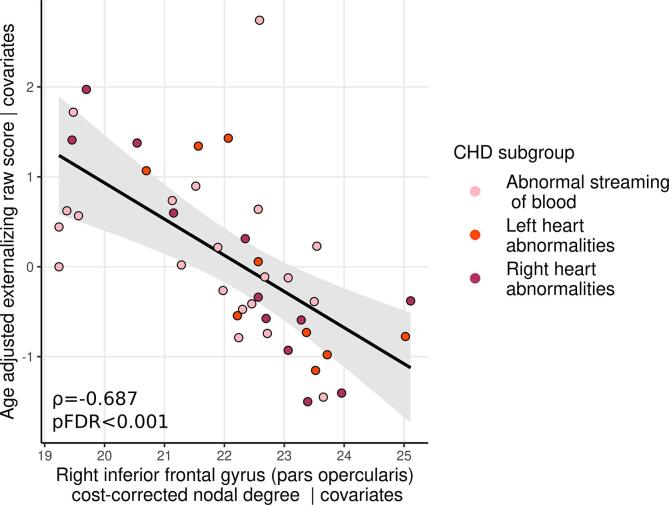
Relationship between cost-corrected nodal degree in the right inferior frontal gyrus (pars opercularis) and age adjusted internalizing raw scores adjusted for covariates.

Partial Spearman’s rank correlations between nodal characteristics and internalizing and externalizing problem scores covarying for GA, postmenstrual age at scan (PMA), sex, brain injury severity, cognitive composite score and IMD are summarized in Fig. 3. Cost-corrected degree in the right inferior frontal gyrus (pars opercularis), a peripheral node, was significantly associated with externalizing scores (partial ρ = −0.687 p_FDR_ < 0.001; Fig. 4). Post-hoc analyses reveal both aggressive (partial ρ = −0.654 p_FDR_ < 0.001) and attention problem subscales (partial ρ = −0.588 p_FDR_ < 0.001) were associated with cost-corrected nodal degree in the right inferior frontal gyrus (pars opercularis).

There were no other significant associations between cost-corrected nodal degree, strength or efficiency and internalizing or externalizing problem scores.

### 3.5 Network based statistics

**Fig. 5:**
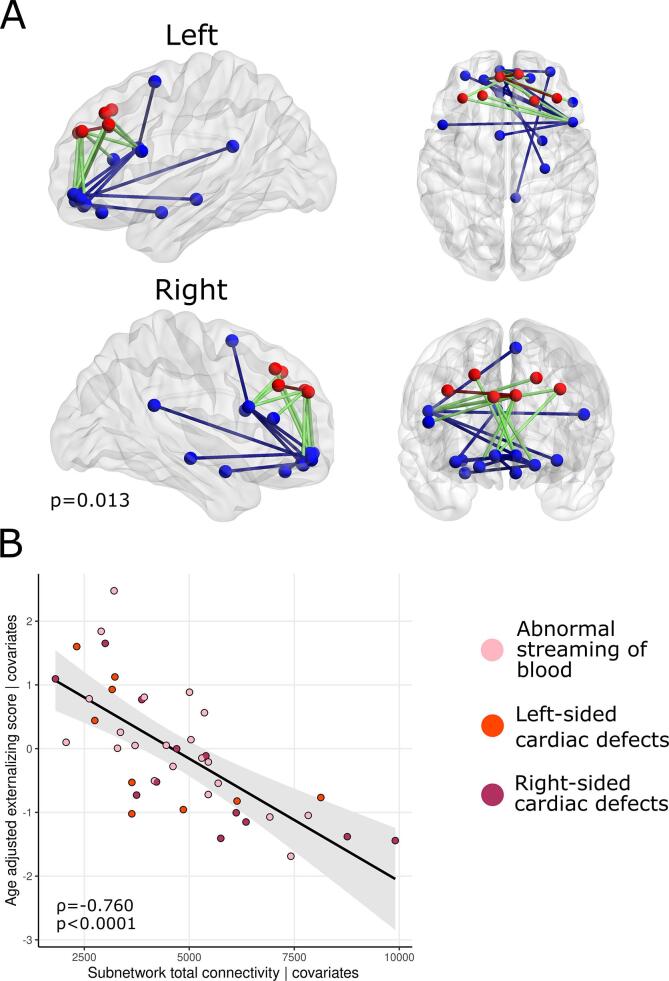
A) Network of reduced connectivity associated with increased externalizing behaviors, red nodes are core, blue nodes are peripheral; red edges are core connections, blue edges are peripheral connections and green edges are peripheral to core connections. B) Association between age adjusted externalizing raw scores and sub-network connectivity from sub-network adjusted for covariates. (For interpretation of the references to colour in this figure legend, the reader is referred to the web version of this article.)

Network based statistics revealed a frontal-limbic sub-network (p = 0.013) of 20 nodes (6 core, 14 peripheral), including the right inferior frontal gyrus (pars opercularis), sharing 25 edges (2 core, 10 feeder and 13 peripheral) where reduced connectivity was associated with higher age adjusted externalizing raw scores in toddlers with CHD covarying for GA, PMA, sex, brain injury severity, cognitive composite score and IMD (Fig. 5). Table 5 lists edges negatively associated with externalizing scores and the associated *t*-statistic at a threshold of *t* = 3.1. Sensitivity analysis revealed no significant edges at *t*-statistic thresholds of 2.5, 2.6 and 2.9. Significant edges were identified in at *t*-statistic thresholds of 2.7, 2.8 and 3.0–3.5 (Supplementary Table 2).

**Table 5:** Nodes and edges where reduced connectivity was significantly associated with higher age adjusted externalizing scores.

**Nodes**		**Edges**		
**Location**	**Type**	**Location**	**Type**	***t*-stat**
Superior frontal gyrus (medial) left	Core	Inferior frontal gyrus (pars opercularis) right - Superior frontal gyrus (medial) left	Feeder	5.02
Superior frontal gyrus (medial) right	Core	Inferior frontal gyrus (pars opercularis) right - Superior frontal gyrus (dorsal) left	Feeder	4.08
Superior frontal gyrus (dorsal) left	Core	Orbitofrontal cortex (medial) left - Orbitofrontal cortex (medial) right	Peripheral	3.7
Superior frontal gyrus (dorsal) right	Core	Inferior frontal gyrus (pars opercularis) right - Inferior frontal gyrus (pars opercularis) left	Peripheral	3.69
Middle frontal gyrus left	Core	Superior frontal gyrus (medial) right - Orbitofrontal cortex (medial) left	Feeder	3.65
Middle frontal gyrus right	Core	Inferior frontal gyrus (pars opercularis) right - Supplementary motor area left	Peripheral	3.65
Rectus gyrus left	Peripheral	Orbitofrontal cortex (medial) right - Rectus gyrus left	Peripheral	3.64
Orbitofrontal cortex (medial) left	Peripheral	Inferior frontal gyrus (pars triangularis) right - Superior frontal gyrus (medial) left	Feeder	3.55
Orbitofrontal cortex (medial) right	Peripheral	Middle frontal gyrus right - Superior frontal gyrus (medial) left	Core	3.52
Orbitofrontal cortex (middle) left	Peripheral	Superior frontal gyrus (medial) left - Orbitofrontal cortex (medial) right	Feeder	3.47
Orbitofrontal cortex (middle) right	Peripheral	Orbitofrontal cortex (middle) right - Posterior cingulate gyrus right	Peripheral	3.36
Orbitofrontal cortex (superior) left	Peripheral	Orbitofrontal cortex (middle) right - Orbitofrontal cortex (medial) left	Peripheral	3.34
Orbitofrontal cortex (superior) right	Peripheral	Superior frontal gyrus (medial) left - Superior frontal gyrus (medial) right	Core	3.32
Inferior frontal gyrus (Opercular) left	Peripheral	Inferior frontal gyrus (pars opercularis) right - Middle frontal gyrus left	Feeder	3.3
Inferior frontal gyrus (Opercular) right	Peripheral	Inferior frontal gyrus (pars opercularis) right - Orbitofrontal cortex (middle) left	Peripheral	3.26
Inferior frontal gyrus (pars triangularis) right	Peripheral	Orbitofrontal cortex (superior) left - Orbitofrontal cortex (medial) right	Peripheral	3.23
Supplementary motor area left	Peripheral	Orbitofrontal cortex (medial) left - Hippocampus right	Peripheral	3.23
Posterior cingulate gyrus right	Peripheral	Inferior frontal gyrus (pars opercularis) right - Orbitofrontal cortex (medial) left	Peripheral	3.2
Hippocampus right	Peripheral	Orbitofrontal cortex (superior) right - Superior frontal gyrus (medial) left	Feeder	3.19
Amygdala Right	Peripheral	Orbitofrontal cortex (superior) left - Amygdala right	Peripheral	3.16
		Orbitofrontal cortex (superior) left - Superior frontal gyrus (medial) right	Feeder	3.16
		Superior frontal gyrus (dorsal) right - Orbitofrontal cortex (medial) left	Feeder	3.15
		Orbitofrontal cortex (superior) right - Orbitofrontal cortex (medial) left	Peripheral	3.13
		Inferior frontal gyrus (pars opercularis) right - Orbitofrontal cortex (superior) left	Peripheral	3.13
		Middle frontal gyrus left - Orbitofrontal cortex (medial) right	Feeder	3.11

Post-hoc partial correlations revealed total connectivity in the sub-network was significantly associated with aggressive (ρ = −0.779 p_FDR_ < 0.001) and attention problems (ρ = −0.607 p_FDR_ < 0.001) subscales.

### 3.6 Global brain network correlates of internalizing and externalizing problem scores

There were no significant relationships between global brain network features and internalizing or externalizing problem scores (Table 6).

**Table 6:** Partial Spearman’s rank correlations between global network features and internalizing and externalizing scores.

	**Age adjusted internalizing raw scores**		**Age adjusted externalizing raw scores**	
**Network Feature**	Partial ρ	p_FDR_	Partial ρ	p_FDR_
Total Network Density	−0.178	0.575	−0.374	0.208
Cost-corrected average nodal strength	−0.235	0.544	−0.066	0.801
Cost-corrected global efficiency	−0.144	0.575	0.016	0.925
Cost-corrected local efficiency	−0.217	0.544	−0.135	0.575

## 4 Discussion

This study investigated the association between neonatal structural brain network organization, clinical variables and internalizing and externalizing symptoms in toddlers with CHD. 28 % of toddlers with CHD had elevated internalizing or externalizing problem scores. Using graph theoretical analyses, we identified associations between reduced frontal-limbic structural connectivity and integration of the right inferior frontal gyrus (pars opercularis) before surgery and higher externalizing symptoms at 22 months. In contrast, increased internalizing symptoms were associated with earlier corrective or final palliative surgery but not brain connectivity.

While mean internalizing and externalizing problem *T*-scores were within the normal range, 28 % of toddlers with CHD had internalizing or externalizing problem scores within the borderline or clinical ranges. This is in keeping with a systematic review which found that while, overall, preschool children with CHD score within the normal ranges, a large subset show increased internalizing or externalizing behaviors compared to healthy peers (Clancy et al., 2020). In addition, children and adolescents with CHD are at increased risk of internalizing disorders such as depression and anxiety (Landolt et al., 2014, Pradhan et al., 1998) and externalizing difficulties such as ADHD, inattention and hyperactivity (Gonzalez et al., 2021, Liamlahi et al., 2014, Shillingford et al., 2008). These findings suggest that from early childhood, children with CHD are at increased risk of emotional and behavioral regulation impairments.

Several studies have implicated altered frontal-limbic networks in externalizing behaviors in both healthy children and clinical populations. In the healthy population, lower neonatal clustering coefficient of the right amygdala was associated with higher externalizing scores at 22 and 48 months, while higher clustering coefficient of the right inferior frontal cortex has been reported to predict higher externalizing symptoms at 48 months (Wee et al., 2017). Higher externalizing behaviors have also been linked to thinner right orbitofrontal cortex (Tanzer et al., 2021), left orbitofrontal cortex and right retrosplenial cortex and reduced structural covariance between the left amgydala and orbitofrontal cortex (Ameis et al., 2014). Lower fractional anisotropy in the left cingulum bundle and uncinate fasciculus (Andre et al., 2020) and increased functional connectivity between the amygdala and orbitofrontal cortex (Thijssen et al., 2021) have been associated with higher externalizing symptoms in healthy children and adolescents. In children with autism, reduced functional connectivity between the amygdala and ventrolateral prefrontal cortex bilaterally was associated with higher externalizing behaviors (Ibrahim et al., 2019). Taken together with our results, this evidence demonstrates the importance of frontal-limbic circuitry for the development of externalizing behaviors in childhood.

Alterations in frontal-limbic circuitry have also been associated with exposure to stressors in early life. Early adverse childhood experiences are associated with altered functional connectivity of the inferior frontal gyrus in childhood (Barch et al., 2018). The degree of altered connectivity predicted the severity of externalizing symptoms over childhood and early adolescence (Barch et al., 2018). In both premature and full-term toddlers, increased functional connectivity between the left striatum and right frontal pole at term/term-equivalent age mediated the relationship between low socioeconomic status and increased externalizing symptoms at age two (Ramphal et al., 2020). In healthy-four-year-old boys, lower fractional anisotropy in fibres connecting the right amygdala and prefrontal cortex mediated the relationship between maternal prenatal depression and increased externalizing behaviors (Hay et al., 2020). CHD may act as an early life stressor, altering frontal-limbic connectivity in the developing brain leading to increased externalizing behaviors in childhood, however this hypothesis requires further investigation.

It has been proposed that the development of attentional control, self-regulation and inhibition in early childhood scaffold the development of more complex executive functions related to cognitive flexibility, goal setting and problem solving (Anderson, 2002, Diamond, 2013). In preschool children with externalizing disorders, impaired executive functions are associated with externalizing behaviors and the strength of association increases with age (Schoemaker et al., 2013). A recent meta-analysis reported children with CHD have impaired executive functions (Feldmann et al., 2021). It is possible that impaired development of the frontal network identified in our study is associated with early externalizing behaviors and subsequent executive function impairments in children with CHD.

To our knowledge, this is the first study to identify an association between younger age at surgery and higher internalizing symptoms in infants who undergo corrective or final palliative surgery in the neonatal period. This contrasts with previously published work reporting improved brain growth and early language abilities in infants with TGA (Lim et al., 2019) and better clinical outcomes in infants with hypoplastic left heart syndrome (Anderson et al., 2015) who underwent earlier cardiac surgery. However, these publications included a different range of cardiac defects compared to this study and investigated different outcome measures; it is therefore not possible to draw direct conclusions. It is also important to note that earlier surgery in the neonatal period may reflect more severe illness which may, in turn, be associated with increased internalizing behaviors in early childhood.

Childhood internalizing symptoms have been linked to changes in DNA methylation (Cimino et al., 2021). In particular, accelerated epigenetic aging has been implicated in internalizing behaviors in healthy children (Tollenaar et al., 2021) and those exposed to maltreatment (Dammering et al., 2021). Early adverse life events alter epigenetic modulation of DNA transcriptional activity (Hyman, 2009). This has been studied in premature infants with high pain exposure, neonatal morbidity and adverse NICU experiences implicated in changes to epigenetic mechanisms such as DNA methylation (Everson et al., 2020, Giarraputo et al., 2017, Provenzi et al., 2015, Provenzi et al., 2018). Altered methylation of serotonin transporter genes at NICU discharge has been associated with temperament changes and poor stress regulation at 3 months in this population (Montirosso et al., 2016a, Montirosso et al., 2016b). In critically ill children, early treatment with parenteral nutrition is associated with increased methylation and subsequent behavioral impairments 4 years post treatment (Jacobs et al., 2021). Altered DNA methylation has been reported in neonates with CHD (Cao et al., 2021, Chang et al., 2021) although to our knowledge the effect of cardiac surgery has yet to be investigated. Earlier cardiac surgery or more severe neonatal illness may induce epigenetic changes associated with increased risk of internalizing symptoms in early childhood, however this hypothesis requires further investigation.

## 5 Limitations

It is important to acknowledge that this work has some limitations. We did not include a control group and therefore cannot determine whether the results are specific to CHD or may also be identified in healthy children. We did not acquire postsurgical imaging in these infants and therefore it was not possible to determine if postsurgical changes in brain connectivity were related to internalizing symptoms at 22 months. Finally, it is possible that in early childhood externalizing symptoms, which manifest in outward behaviors, may be easier to detect than internalizing symptoms (Papachristou and Flouri, 2020). However, externalizing *T*-scores were not significantly higher than internalizing *T*-scores in this cohort [t(42) = 1.43, p = 0.16].

## 6 Conclusions

Toddlers with CHD are at risk of elevated internalizing and externalizing symptoms in early childhood. We provide the first evidence that reduced neonatal structural connectivity of a frontal-limbic network and integration of the right inferior frontal gyrus (pars opercularis) before surgery are associated with increased externalizing symptoms at 22 months in toddlers with CHD. These results complement previous studies in healthy children which have implicated frontal-limbic networks in externalizing behaviours. On the other hand, earlier corrective or final palliative surgery in the neonatal period was associated with higher internalizing symptoms. Frontal-limbic network development and age at surgery may represent important neonatal antecedents of behavioral impairments in CHD in childhood.

## CRediT authorship contribution statement

**Alexandra F. Bonthrone:** Formal analysis, Investigation, Visualization, Methodology, Resources, Data curation, Writing – original draft. **Andrew Chew:** Investigation, Resources, Writing – review & editing. **Megan Ní Bhroin:** Formal analysis, Investigation, Methodology, Writing – review & editing. **Francesca Morassutti Rech:** Investigation, Writing – review & editing. **Christopher J. Kelly:** Methodology, Resources, Data curation, Writing – review & editing. **Daan Christiaens:** Methodology, Software, Writing – review & editing. **Maximilian Pietsch:** Methodology, Software, Writing – review & editing. **J-Donald Tournier:** Methodology, Software, Writing – review & editing. **Lucilio Cordero-Grande:** Methodology, Software, Writing – review & editing. **Anthony Price:** Methodology, Resources, Writing – review & editing. **Alexia Egloff:** Investigation, Writing – review & editing. **Joseph V. Hajnal:** Methodology, Software, Writing – review & editing. **Kuberan Pushparajah:** Validation, Writing – review & editing. **John Simpson:** Validation, Writing – review & editing. **A. David Edwards:** Validation, Writing – review & editing. **Mary A. Rutherford:** Validation, Writing – review & editing. **Chiara Nosarti:** Conceptualization, Methodology, Writing – review & editing. **Dafnis Batalle:** Conceptualization, Methodology, Supervision, Writing – review & editing. **Serena J. Counsell:** Supervision, Conceptualization, Methodology, Visualization, Funding acquisition, Project administration, Writing – original draft.

## Declaration of Competing Interest

The authors declare that they have no known competing financial interests or personal relationships that could have appeared to influence the work reported in this paper.

## Data Availability

Data will be made available on request.
