# Supplementary material for: Neonatal frontal-limbic connectivity is associated with externalizing behaviours in toddlers with Congenital Heart Disease
Source: Neuroimage Clin. 2022 Aug 17;36:103153. doi: 10.1016/j.nicl.2022.103153 (PMC9403726; doi:10.1016/j.nicl.2022.103153)
Supplement: Supplementary data 1 [file mmc1.docx]

| **Supplementary Table 1**. Results of core-periphery partition | | |
| --- | --- | --- |
| **Number** | **Node** | **Classification** |
| 1-2 | Left and right superior frontal gyrus (dorsal) | Core |
| 3-4 | Left and right superior frontal gyrus (medial) | Core |
| 5-6 | Left and right middle frontal gyrus | Core |
| 7-8 | Left and right precentral gyrus | Core |
| 9-10 | Left and right anterior cingulate gyrus | Core |
| 11-12 | Left and right median cingulate and paracingulate gyrus | Core |
| 13-14 | Left and right insula | Core |
| 15-16 | Left and right middle temporal gyrus | Core |
| 17-18 | Left and right precuneus | Core |
| 19-20 | Left and right caudate nucleus | Core |
| 21-22 | Left and right putamen | Core |
| 23-24 | Left and right thalamus | Core |
| 25-26 | Left and right cerebellum | Core |
| 27 | Left inferior frontal gyrus (pars triangularis) | Core |
| 28 | Left inferior parietal lobule | Core |
| 29 | Left calcarine cortex | Core |
| 30 | Left middle occipital cortex | Core |
| 31 | Right angular gyrus | Core |
| 32 | Right fusiform gyrus | Core |
| 33 | Right inferior temporal gyrus | Core |
| 34 | Cerebellar vermis | Core |
| 1-2 | Left and right orbitofrontal cortex (superior) | Peripheral |
| 3-4 | Left and right orbitofrontal cortex (middle) | Peripheral |
| 5-6 | Left and right orbitofrontal cortex (inferior) | Peripheral |
| 7-8 | Left and right orbitofrontal cortex (medial) | Peripheral |
| 9-10 | Left and right gyrus rectus | Peripheral |
| 11-12 | Left and right inferior frontal gyrus (pars opercularis) | Peripheral |
| 13-14 | Left and right supplementary motor area | Peripheral |
| 15-16 | Left and right paracentral lobule | Peripheral |
| 17-18 | Left and right postcentral gyrus | Peripheral |
| 19-20 | Left and right superior parietal cortex | Peripheral |
| 21-22 | Left and right supramarginal gyrus | Peripheral |
| 23-24 | Left and right cuneus | Peripheral |
| 25-26 | Left and right rolandic operculum | Peripheral |
| 27-28 | Left and right Heschl’s gyrus | Peripheral |
| 29-30 | Left and right superior temporal gyrus | Peripheral |
| 31-32 | Left and right temporal pole (superior) | Peripheral |
| 33-34 | Left and right temporal pole (middle) | Peripheral |
| 35-36 | Left and right amygdala | Peripheral |
| 37-38 | Left and right hippocampus | Peripheral |
| 39-40 | Left and right parahippocampal gyrus | Peripheral |
| 41-42 | Left and right olfactory cortex | Peripheral |
| 43-44 | Left and right posterior cingulate gyrus | Peripheral |
| 45-46 | Left and right superior occipital cortex | Peripheral |
| 47-48 | Left and right inferior occipital cortex | Peripheral |
| 49-50 | Left and right lingual gyrus | Peripheral |
| 51-52 | Left and right pallidum | Peripheral |
| 53 | Left angular gyrus | Peripheral |
| 54 | Left fusiform gyrus | Peripheral |
| 55 | Left inferior temporal gyrus | Peripheral |
| 56 | Right inferior frontal gyrus (pars triangularis) | Peripheral |
| 57 | Right calcarine cortex | Peripheral |
| 58 | Right inferior parietal lobule | Peripheral |
| 59 | Right middle occipital cortex | Peripheral |

| **Supplementary Table 2.** Significant edges and corresponding *t*-statistic where reduced connectivity is associated with increased age adjusted externalizing scores at different *t*-statistic thresholds | | | | | | | |
| --- | --- | --- | --- | --- | --- | --- | --- |
| **Edge** | ***t-*statistic threshold** | | | | | | |
|  | ***2.7*** | ***2.8*** | ***3.0*** | ***3.2*** | ***3.3*** | ***3.4*** | ***3.5*** |
| Inferior frontal gyrus (opercular) right - Superior frontal gyrus (medial) left | 5.02 | 5.02 | 5.02 | 5.02 | 5.02 | 5.02 | 5.02 |
| Superior frontal gyrus (dorsal) left - Inferior frontal gyrus (opercular) right | 4.08 | 4.08 | 4.08 | 4.08 | 4.08 | 4.08 | 4.08 |
| Posterior cingulate gyrus left - Postcentral gyrus right | 3.92 | 3.92 | - | - | - | - | - |
| Olfactory cortex right - Posterior cingulate gyrus left | 3.78 | 3.78 | - | - | - | - | - |
| Rolandic operculum left - Cerebellum vermis | 3.72 | 3.72 | - | - | - | - | - |
| Orbitofrontal cortex (medial) left- Orbitofrontal cortex (medial) right | 3.7 | 3.7 | 3.7 | 3.7 | 3.7 | 3.7 | - |
| Inferior frontal gyrus (opercular) left - Inferior frontal gyrus (opercular) right | 3.69 | 3.69 | 3.69 | 3.69 | 3.69 | 3.69 | 3.69 |
| Inferior frontal gyrus (opercular) right - Supplementary motor area left | 3.65 | 3.65 | 3.65 | 3.65 | 3.65 | 3.65 | 3.65 |
| Superior frontal gyrus (medial) right - Orbitofrontal cortex (medial) left | 3.65 | 3.65 | 3.65 | 3.65 | 3.65 | 3.65 | - |
| Orbitofrontal cortex (medial) right - Gyrus rectus left | 3.64 | 3.64 | 3.64 | 3.64 | 3.64 | 3.64 | - |
| Inferior frontal gyrus (pars triangularis) right - Superior frontal gyrus (medial) left | 3.55 | 3.55 | 3.55 | 3.55 | 3.55 | 3.55 | 3.55 |
| Middle frontal gyrus right - Superior frontal gyrus (medial) left | 3.52 | 3.52 | 3.52 | 3.52 | 3.52 | 3.52 | 3.52 |
| Superior frontal gyrus (medial) left - Orbitofrontal cortex (medial) right | 3.47 | 3.47 | 3.47 | 3.47 | 3.47 | 3.47 | - |
| Gyrus rectus right - Thalamus right | 3.43 | - | - | - | - | - | - |
| Precentral gyrus right - Posterior cingulate gyrus left | 3.37 | 3.37 | - | - | - | - | - |
| Orbitofrontal cortex (middle) right - Posterior cingulate gyrus right | 3.36 | 3.36 | 3.36 | 3.36 | 3.36 | - | - |
| Orbitofrontal cortex (middle) right - Orbitofrontal cortex (medial) left | 3.34 | 3.34 | 3.34 | 3.34 | 3.34 | - | - |
| Superior frontal gyrus (medial) left - Superior frontal gyrus (medial) right | 3.32 | 3.32 | 3.32 | 3.32 | 3.32 | - | - |
| Middle frontal gyrus left - Inferior frontal gyrus (opercular) right | 3.3 | 3.3 | 3.3 | 3.3 | - | - | - |
| Orbitofrontal cortex (middle) left - Inferior frontal gyrus (opercular) right | 3.26 | 3.26 | 3.26 | 3.26 | - | - | - |
| Orbitofrontal cortex (superior) left - Orbitofrontal cortex (medial) right | 3.23 | 3.23 | 3.23 | 3.23 | - | - | - |
| Orbitofrontal cortex (medial) left- Hippocampus right | 3.23 | 3.23 | 3.23 | 3.23 | - | - | - |
| Olfactory cortex right - Lingual gyrus right | 3.22 | 3.22 | - | - | - | - | - |
| Inferior frontal gyrus (opercular) right - Orbitofrontal cortex (medial) left | 3.2 | 3.2 | 3.2 | - | - | - | - |
| Orbitofrontal cortex (superior) right - Superior frontal gyrus (medial) left | 3.19 | 3.19 | 3.19 | - | - | - | - |
| Orbitofrontal cortex (superior) left - Superior frontal gyrus (medial) right | 3.16 | 3.16 | 3.16 | - | - | - | - |
| Orbitofrontal cortex (superior) left - Amygdala right | 3.16 | 3.16 | 3.16 | - | - | - | - |
| Superior frontal gyrus (dorsal) right - Orbitofrontal cortex (medial) left | 3.15 | 3.15 | 3.15 | - | - | - | - |
| Orbitofrontal cortex (superior) left - Inferior frontal gyrus (opercular) right | 3.13 | 3.13 | 3.13 | - | - | - | - |
| Orbitofrontal cortex (superior) right - Orbitofrontal cortex (medial) left | 3.13 | 3.13 | 3.13 | - | - | - | - |
| Middle frontal gyrus left - Orbitofrontal cortex (medial) right | 3.11 | 3.11 | 3.11 | - | - | - | - |
| Rolandic operculum left - Precuneus left | 3.1 | 3.1 | - | - | - | - | - |
| Superior frontal gyrus (medial) left - Heschl's gyrus right | 3.05 | 3.05 | 3.05 | - | - | - | - |
| Orbitofrontal cortex (middle) left - Superior frontal gyrus (medial) right | 3.01 | 3.01 | 3.01 | - | - | - | - |
| Posterior cingulate gyrus left - Supramarginal gyrus right | 3.01 | 3.01 | - | - | - | - | - |
| Middle frontal gyrus right - Orbitofrontal cortex (middle) left | 2.97 | 2.97 | - | - | - | - | - |
| Superior frontal gyrus (dorsal) left - Orbitofrontal cortex (medial) right | 2.96 | 2.96 | - | - | - | - | - |
| Pallidum right - Cerebellum vermis | 2.96 | 2.96 | - | - | - | - | - |
| Orbitofrontal cortex (middle) left - Orbitofrontal cortex (medial) right | 2.95 | 2.95 | - | - | - | - | - |
| Supplementary motor area right - Posterior cingulate gyrus left | 2.93 | 2.93 | - | - | - | - | - |
| Orbitofrontal cortex (superior) left - Inferior frontal gyrus (pars triangularis) right | 2.92 | 2.92 | - | - | - | - | - |
| Posterior cingulate gyrus right - Cerebellum left | 2.92 | 2.92 | - | - | - | - | - |
| Orbitofrontal cortex (superior) left - Middle frontal gyrus right | 2.9 | 2.9 | - | - | - | - | - |
| Posterior cingulate gyrus left - Cerebellum vermis | 2.9 | 2.9 | - | - | - | - | - |
| Posterior cingulate gyrus left - Caudate right | 2.88 | 2.88 | - | - | - | - | - |
| Precentral gyrus left - Inferior frontal gyrus (opercular) right | 2.87 | 2.87 | - | - | - | - | - |
| Superior frontal gyrus (medial) right - Gyrus rectus left | 2.87 | 2.87 | - | - | - | - | - |
| Posterior cingulate gyrus left - Heschl's gyrus right | 2.84 | 2.84 | - | - | - | - | - |
| Inferior frontal gyrus (opercular) right - Supplementary motor area right | 2.81 | 2.81 | - | - | - | - | - |
| Superior frontal gyrus (dorsal) left - Inferior frontal gyrus (pars triangularis) right | 2.8 | - | - | - | - | - | - |
| Orbitofrontal cortex (superior) left - Temporal pole (superior) right | 2.79 | - | - | - | - | - | - |
| Inferior frontal gyrus (opercular) right - Inferior frontal gyrus (pars triangularis) left | 2.78 | - | - | - | - | - | - |
| Orbitofrontal cortex (middle) right - Gyrus rectus left | 2.77 | - | - | - | - | - | - |
| Inferior frontal gyrus (opercular) right - Thalamus right | 2.77 | - | - | - | - | - | - |
| Posterior cingulate gyrus right - Heschl's gyrus right | 2.77 | - | - | - | - | - | - |
| Inferior frontal gyrus (opercular) right - Superior frontal gyrus (medial) right | 2.76 | - | - | - | - | - | - |
| Middle frontal gyrus right - Orbitofrontal cortex (medial) left | 2.76 | - | - | - | - | - | - |
| Inferior frontal gyrus (opercular) right - Precuneus left | 2.76 | - | - | - | - | - | - |
| Superior frontal gyrus (medial) left - Occipital cortex (superior) right | 2.74 | - | - | - | - | - | - |
| Posterior cingulate gyrus left - Superior temporal gyrus right | 2.74 | - | - | - | - | - | - |
| Orbitofrontal cortex (middle) right - Superior frontal gyrus (medial) left | 2.72 | - | - | - | - | - | - |
| Orbitofrontal cortex (medial) left- Gyrus rectus right | 2.71 | - | - | - | - | - | - |
| Orbitofrontal cortex (medial) left- Lingual gyrus right | 2.71 | - | - | - | - | - | - |
